# Design, Synthesis, Biological Evaluation and Molecular Modeling Study of Novel Indolizine-1-Carbonitrile Derivatives as Potential Anti-Microbial Agents

**Published:** 2018

**Authors:** Ehsan Faghih-Mirzaei, Mohammad Seifi, Mehdi Abaszadeh, Kamiar Zomorodian, Helia Helali

**Affiliations:** a *Department of Medicinal Chemistry, Faculty of Pharmacy, Kerman University of Medical Sciences, Kerman, Iran. *; b *Pharmaceutics Research Center, Institute of Neuropharmacology, Kerman University of Medical Sciences, Kerman, Iran.*; c *Department of Chemistry, Faculty of Sciences, Islamic Azad University of Najafabad, Najafabad, Isfahan, Iran.*; d *Department of Medical Mycology and Parasitology, Basic Sciences in Infectious Diseases Research Center, School of Medicine, Shiraz University of Medical Sciences, Shiraz, Iran.*

**Keywords:** Indolizine-1-carbonitriles; 1, 3-Dipolar cycloaddition, Ultrasound irradiation, Antifungal activity, Antibacterial activity, Molecular docking

## Abstract

A novel one-pot two step tandem reaction for the synthesis of indolizine-1-carbonitrile derivatives (5a-i) was identified. The route comprises 1,3-dipolar cycloaddition reaction of aromatic aldehyde derivatives (1a-i), malononitrile (2) and 1-(2-(4-bromophenyl)-2-oxoethyl)-2-chloropyridin-1-ium (4) under ultrasound irradiation at room temperature in the presence of triethylamine at acetonitrile. The product compounds were tested against bacteria and fungi. It was revealed that compound 5b had the most antifungal activity (range MICs = 8–32 µg/mL) and compound 5g had the most antibacterial activity (range MICs = 16–256 µg/mL). Molecular docking of compounds (5a-i) into fungal 14α-demethylase and bacterial protein tyrosine phosphatase active sites were also performed and probable binding mode of compounds 5b and 5g were determined.

## Introduction

Invasive bacterial and fungal infections are well recognized as diseases of immuno-compromised patients. Over the last twenty years, there have been significant increase in the number of recorded instances of resistant bacterial and fungal infections (1). It has been prospected that in the year 2050, the number of mortality due to microbial resistance will surpass mortality due to cancer (2). Thus, the discovery of novel antimicrobial agents is crucial in coming years and is required to guarantee positive therapeutic outcomes in patients. 

Among N-containing heterocyclic compounds, indolizine and its derivatives have an important heterocyclic nucleus with diverse biological activities such as anticancer (3), antitubercular (4), antioxidant (5), antimicrobial (6), analgesic (7) and anti-inflammatory (8) activities. This versatile nature of the indolizine derivatives makes them a good candidate for the synthesis of new antimicrobial agents (9). In the last few years, a number of indolizine derivatives with antibacterial and antifungal effects have been developed (9). Some of the indolizine-1-carbonitrile derivatives have been proved to be potent inhibitors of the bacterial protein tyrosine phosphatase (PTPs) (10) which are essential for the virulence of several pathogenic bacteria (11-13). In addition, some indolizine derivatives have been shown to exhibit dual antifungal and antibacterial activity against gram-negative and positive bacteria, as well as fungi including *Candida albicans *and* Aspergillusflavus *(14).

Due to the structural resemblance to azole antifungal, indolizine and its derivatives can also serve as antifungal agents (15). Azole antifungals inhibit the lanosterol 14α-demethylase (CYP51), the key enzyme in ergosterol biosynthesis from lanosterol in yeast and fungi. The selective inhibition of CYP51 causes depletion of ergosterol, accumulation of lanosterol and other 14α-methyl sterols, resulting in the growth inhibition of fungal cells (16). However, resistance to azoles has been widely reported and is now becoming a serious clinical problem (17). Therefore, the synthesis of novel compounds capable of inhibiting 14α-demethylase is of great interest. 

**Scheme 1:**
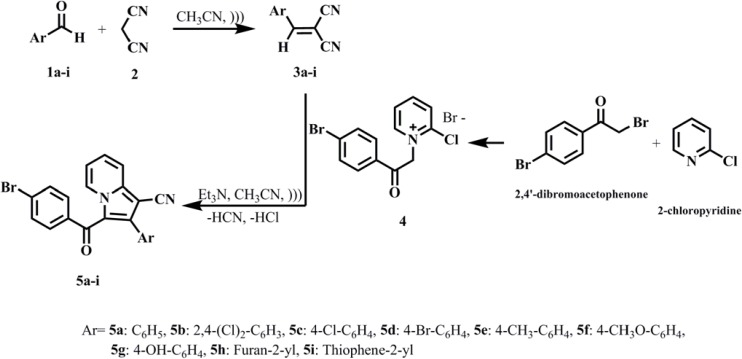
Synthesis of indolizine-1-carbonitrile derivatives

Ultrasound irradiation is one of the most significant techniques, increasingly used as a green synthetic approach for accelerating organic chemical reactions (18-20). The main advantages of this method compared to common laboratory techniques are shortening of reaction completion, increased reaction efficiency, high purity of main products and of course lessening by products generation, reaction execution under moderate conditions and improved selectivity (21-23). In the synthesis part of this project, a novel one-pot two-step tandem reaction was designed for the synthesis of indolizine-1-carbonitrile derivatives (5a-i) using 1,3-dipolar cycloaddition reaction of aromatic aldehyde derivatives (1a-i), malononitrile (2) and 1-(2-(4-bromophenyl)-2-oxoethyl)-2-chloropyridin-1-ium (4) under ultrasound irradiation at room temperature in the presence of triethylamine at acetonitrile (Scheme 1). Although, a major part of the research on newly synthesized indolizine derivatives has focused on anticancer drug discovery (24-26), the emphasis in this study was directed at identifying new indolizine compounds with improved antibacterial and antifungal properties. In this study, synthesis combined with computational docking studies and biological screening assays, led to the identification of indolizine-1-carbonitrile 5b, a compound with potent *in-vitro* antifungal activity and compounds 5h and 5g with moderate antibacterial activity.

## Experimental

*Chemistry*

Solvents and reagents were purchased from commercial sources and were used without further purification. Melting points were measured on an Electrothermal-9100 apparatus and are uncorrected. IR spectra were recorded on a Brucker FTIR Tensor 27 infrared spectrophotometer. ^1^H NMR spectra were recorded on a BruckerAvance III 400 MHz spectrometer. ^13^C NMR spectra were recorded on the same instruments at 100 MHz, using TMS as an internal standard. Mass spectra were measured on a GCMS-QP1000 EX spectrometer at 70 eV. Elemental analyses were performed using a Heraeus CHN-O-Rapid analyzer. Ultrasonication was performed in a Bandelin SONOREX^TM^ Ultrasonic Bath (Super RK) at a frequency of 100 kHz. The internal dimensions of the ultrasonic cleaner tank were 240 × 140 × 100 mm with liquid holding capacity of 3 L.

*General procedure for the preparation of compounds 5a-i*

A solution of aldehyde derivatives (1a-i) (2 mmol) and malononitrile (2) (2 mmol) in acetonitrile (10 mL) was ultrasound irradiated at room temperature for about 5 min. 1-(2-(4-bromophenyl)-2-oxoethyl)-2-chloropyridin-1-ium bromide (4) (2 mmol) and triethylamine (2 mmol) were added to the reaction mixture, and irradiated with ultrasound at room temperature for about 20-30 min (the progress of the reaction was monitored by TLC and n-hexane/ethyl acetate was used as eluent). The solvent was diluted with 50 mL of water and the resulting precipitate was collected by filtration. The crude product was recrystallized with dichloromethane/n-hexane (1:2), yielding a pure solid sample for analysis.

*3-(4-Bromobenzoyl)-2-phenylindolizine-1-carbonitrile* *(5a)*

Yellow crystals, yield: 93%. m.p. 180 °C (dec.). IR (KBr, *ν*_max_/cm^-1^): 2192 (CN), 1648 (C=O), 1616, 1580 (C=C). ^1^H NMR (400 MHz, CDCl_3_) δ_ppm_: 7.95 (t, 1H, ^3^J = 4Hz, CH-Ar), 7.93 (t, 1H, ^3^J = 4Hz, CH-Ar), 7.81 (s, 1H, CH-Ar), 7.72 (t, 1H, ^3^J = 4Hz, CH-Ar), 7.70 (t, 1H, ^3^J = 4Hz, CH-Ar), 7.53-722 (m, 6H, CH-Ar), 7.95 (t, 1H, ^3^J = 4Hz, CH-Ar), 6.64-6.60 (m, 1H, CH-Ar). ^13^C NMR (100 MHz, CDCl_3_) δ_ppm_: 188.71 (C=O), 156.08, 145.20, 141.66, 140.34, 137.96, 136.48, 134.49, 132.71, 132.13, 131.48, 130.73, 130.20, 129.74, 127.93, 122.51, 117.63, 113.35 (CN). MS (*m/z*): 402 (M + 2) (10), 400 (M^+^‏) (11), 340 (5), 287 (10), 244 (100), 217 (14), 185 (26), 157 (23), 129 (8), 102 (5), 76 (10). Anal.calcd. for C_22_H_13_BrN_2_O: C, 65.85; H, 3.27; N, 6.98%. Found: C, 65.67; H, 3.13; N, 6.79%.

*3-(4-Bromobenzoyl)-2-(2,4-dichlorophenyl)indolizine-1-carbonitrile (5b)*

Yellow crystals, yield: 91%. m.p. 94 °C (dec.). IR (KBr, *ν*_max_/cm^-1^): 2208 (CN), 1657 (C=O), 1577, 1536 (C=C). ^1^H NMR (400 MHz, CDCl_3_) δ_ppm_: 8.10 (s, 1H, CH-Ar), 7.98 (d, 2H, ^3^J = 8Hz, CH-Ar), 7.72 (d, 2H, ^3^J = 8Hz, CH-Ar), 7.55-6.92 (m, 5H, CH-Ar), 6.58-6.54 (m, 1H, CH-Ar). ^13^C NMR (100 MHz, CDCl_3_) δ_ppm_: 187.72 (C=O), 156.59, 140.05, 139.70, 139.15, 138.72, 137.94, 133.74, 132.82, 132.21, 131.70, 130.58, 130.29, 128.54, 128.47, 127.52, 122.38, 113.40 (CN). MS (*m/z*): 470 (M + 2) (3), 468 (M^+^‏) (3.3), 463 (16), 461 (16.6), 435 (18), 355 (34), 312 (100), 277 (48), 241 (27), 185 (96), 143 (73), 105 (6), 76 (22). Anal.calcd. for C_22_H_11_BrCl_2_N_2_O: C, 56.20; H, 2.36; N, 5.96%. Found: C, 55.98; H, 2.19; N, 5.77%.

*3-(4-Bromobenzoyl)-2-(4-chlorophenyl)indolizine-1-carbonitrile (5c)*

Yellow crystals, yield: 90%. m.p. 105 °C (dec.). IR (KBr, *ν*_max_/cm^-1^): 2192 (CN), 1651 (C=O), 1580, 1539 (C=C). ^1^H NMR (400 MHz, CDCl_3_) δ_ppm_: 7.93 (t, 1H, ^3^J = 4Hz, CH-Ar), 7.91 (t, 1H, ^3^J = 4Hz, CH-Ar), 7.71 (d, 2H, ^3^J = 8Hz, CH-Ar), 7.50-7.49 (m, 1H, CH-Ar), 7.41 (d, 2H, ^3^J = 8Hz, CH-Ar), 7.17 (d, 2H, ^3^J = 8Hz, CH-Ar), 7.02 (d, 2H, ^3^J = 8Hz, CH-Ar), 6.65-6.61 (m, 1H, CH-Ar). ^13^C NMR (100 MHz, CDCl_3_) δ_ppm_: 188.49 (C=O), 156.00, 143.64, 140.08, 139.12, 137.98, 136.70, 134.34, 132.19, 131.79, 131.43, 130.15, 128.86, 128.61, 128.09, 122.59, 117.45, 113.41 (CN). MS (*m/z*): 436 (M + 2) (11), 434 (M^+^‏) (12), 366 (5), 321 (15), 278 (100), 243 (29), 215 (18), 185 (44), 143 (41), 114 (5), 75 (18). Anal.calcd. for C_22_H_12_BrClN_2_O: C, 60.65; H, 2.78; N, 6.43%. Found: C, 60.47; H, 2.59; N, 6.28%.

*3-(4-Bromobenzoyl)-2-(4-bromophenyl)indolizine-1-carbonitrile (5d)*

Green crystals, yield: 89%. m.p. 95 °C (dec.). IR (KBr, *ν*_max_/cm^-1^): 2208 (CN), 1654 (C=O), 1584, 1539 (C=C). ^1^H NMR (400 MHz, CDCl_3_) δ_ppm_: 7.92 (t, 1H, ^3^J = 4Hz, CH-Ar), 7.70 (t, 1H, ^3^J = 4Hz, CH-Ar), 7.58-7.49 (m, 5H, CH-Ar), 7.30 (d, 2H, ^3^J = 8Hz, CH-Ar), 7.09 (d, 2H, ^3^J = 8Hz, CH-Ar), 6.92-6.88 (m, 1H, CH-Ar). ^13^C NMR (100 MHz, CDCl_3_) δ_ppm_: 188.49 (C=O), 155.97, 143.76, 140.04, 138.00, 133.13, 132.73, 132.20, 131.85, 131.44, 129.64, 129.12, 128.69, 123.54, 122.60, 122.00, 117.78, 113.43 (CN). MS (*m/z*): 482 (M + 4) (9), 480 (M + 2) (17), 478 (M^+^‏) (10), 341 (99), 311 (19), 259 (33), 216 (23), 185 (100), 157 (84), 129 (51), 76 (72). Anal.calcd. for C_22_H_12_Br_2_N_2_O: C, 55.03; H, 2.52; N, 5.83%. Found: C, 54.82; H, 2.36; N, 5.67%.

*3-(4-Bromobenzoyl)-2-(p-tolyl)indolizine-1-carbonitrile (5e)*

Yellow crystals, yield: 88%. m.p. 255-257 °C. IR (KBr, *ν*_max_/cm^-1^): 2192 (CN), 1654 (C=O), 1596, 1580 (C=C). ^1^H NMR (400 MHz, DMSO-d_6_) δ_ppm_: 7.98-7.84 (m, 6H, CH-Ar), 7.40 (t, 1H, ^3^J = 4Hz, CH-Ar), 7.28 (d, 2H, ^3^J = 8Hz, CH-Ar), 7.12 (d, 2H, ^3^J = 8Hz, CH-Ar), 7.00 (t, 1H, ^3^J = 4Hz, CH-Ar), 2.33 (s, 3H, CH_3_). ^13^C NMR (100 MHz, DMSO-d_6_) δ_ppm_: 189.42 (C=O), 154.68, 145.16, 143.29, 141.72, 140.58, 135.02, 134.31, 132.25, 131.66, 131.51, 130.79, 130.18, 127.77, 126.26, 120.94, 118.08, 114.82 (CN), 21.11 (CH_3_). MS (*m/z*): 416 (M + 2) (7), 414 (M^+^‏) (8), 340 (4), 301 (25), 258 (100), 231 (11), 185 (30), 143 (30), 115 (13), 76 (8). Anal.calcd. for C_23_H_15_BrN_2_O: C, 66.52; H, 3.64; N, 6.75%. Found: C, 66.36; H, 3.48; N, 6.57%.

*3-(4-Bromobenzoyl)-2-(4-methoxyphenyl)indolizine-1-carbonitrile (5f)*

Green crystals, yield: 86%. m.p. 221-224 °C. IR (KBr, *ν*_max_/cm^-1^): 2192 (CN), 1651 (C=O), 1590, 1558 (C=C). ^1^H NMR (400 MHz, DMSO-d_6_) δ_ppm_: 7.98-7.83 (m, 6H, CH-Ar), 7.41 (t, 1H, ^3^J = 4Hz, CH-Ar), 7.19 (d, 2H, ^3^J = 8Hz, CH-Ar), 7.05-6.99 (m, 3H, CH-Ar), 3.81 (s, 3H, OCH_3_). ^13^C NMR (100 MHz, DMSO-d_6_) δ_ppm_: 189.36 (C=O), 162.76, 160.46, 154.66, 144.80, 141.88, 140.59, 135.29, 133.27, 132.92, 131.62, 131.43, 126.03, 122.88, 120.95, 118.14, 115.25, 114.95 (CN), 55.63 (OCH_3_). MS (*m/z*): 432 (M + 2) (43), 430 (M^+^‏) (44), 339 (100), 315 (71), 277 (88), 231 (42), 185 (65), 157 (83), 129 (25), 104 (19), 78 (49). Anal.calcd. for C_23_H_15_BrN_2_O_2_: C, 64.05; H, 3.51; N, 6.50%. Found: C, 63.82; H, 3.38; N, 6.33%.

*3-(4-Bromobenzoyl)-2-(4-hydroxyphenyl)indolizine-1-carbonitrile (5g)*

Yellow crystals, yield: 85%. m.p. 112 °C (dec.). IR (KBr, *ν*_max_/cm^-1^): 3568 (broad, OH), 2192 (CN), 1651 (C=O), 1584, 1542 (C=C). ^1^H NMR (400 MHz, DMSO-d_6_) δ_ppm_: 7.94 (t, 1H, ^3^J = 4Hz, CH-Ar), 7.87-7.39 (m, 6H, CH-Ar), 7.06-6.98 (m, 3H, CH-Ar), 6.79 (d, 2H, ^3^J = 8Hz, CH-Ar), 3.48 (s, broad, OH). ^13^C NMR (100 MHz, DMSO-d_6_) δ_ppm_: 189.21 (C=O), 162.62, 154.71, 145.11, 142.06, 140.51, 135.56, 133.77, 132.79, 131.90, 131.57, 131.33, 127.61, 125.80, 120.92, 118.20, 116.80, 114.94 (CN). MS (*m/z*): 418 (M + 2) (4.5), 416 (M^+^‏) (4.9), 341 (100), 259 (28), 231 (16), 185 (38), 157 (41), 129 (30), 78 (19). Anal.calcd. for C_22_H_13_BrN_2_O_2_: C, 63.33; H, 3.14; N, 6.71%. Found: C, 63.11; H, 2.97; N, 6.52%.

*3-(4-Bromobenzoyl)-2-(furan-2-yl)indolizine-1-carbonitrile (5h)*

Yellow crystals, yield: 87%. m.p. 248-250 °C. IR (KBr, *ν*_max_/cm^-1^): 2176 (CN), 1654 (C=O), 1580, 1542 (C=C). ^1^H NMR (400 MHz, DMSO-d_6_) δ_ppm_: 8.01-7.78 (m, 8H, CH-Ar), 7.38 (d, 1H, ^3^J = 4Hz, CH-Ar), 7.28 (d, 1H, ^3^J = 4Hz, CH-Ar), 6.99 (t, 1H, ^3^J = 4Hz, CH-Ar). ^13^C NMR (100 MHz, DMSO-d_6_) δ_ppm_: 188.97 (C=O), 155.18, 150.49, 146.93, 142.28, 140.33, 135.26, 131.61, 131.23, 131.08, 130.50, 125.88, 124.68, 120.56, 118.53, 114.14, 114.06 (CN). MS (*m/z*): 392 (M + 2) (28), 390 (M^+^‏) (30), 340 (6), 277 (40), 234 (100), 185 (41), 143 (44), 114 (5), 76 (12). Anal.calcd. for C_20_H_11_BrN_2_O_2_: C, 61.40; H, 2.83; N, 7.16%. Found: C, 61.19; H, 2.70; N, 6.98%.

*3-(4-Bromobenzoyl)-2-(thiophen-2-yl)indolizine-1-carbonitrile (5i)*

Yellow crystals, yield: 86%. m.p. 249-251 °C. IR (KBr, *ν*_max_/cm^-1^): 2176 (CN), 1651 (C=O), 1596, 1539 (C=C). ^1^H NMR (400 MHz, DMSO-d_6_) δ_ppm_: 8.25-7.80 (m, 8H, CH-Ar), 7.45 (d, 1H, ^3^J = 4Hz, CH-Ar), 7.31 (t, 1H, ^3^J = 4Hz, CH-Ar), 7.07 (d, 1H, ^3^J = 4Hz, CH-Ar). ^13^C NMR (100 MHz, DMSO-d_6_) δ_ppm_: 189.07 (C=O), 154.82, 150.27, 141.71, 140.80, 140.07, 138.51, 137.52, 135.14, 133.54, 131.63, 131.32, 128.85, 125.96, 121.41, 118.08, 115.44 (CN). MS (*m/z*): 408 (M + 2) (9.2), 406 (M^+^‏) (9.7), 340 (2.7), 338 (3.2), 293 (51), 250 (100), 223 (11), 185 (40), 143 (63), 108 (10), 76 (25). Anal.calcd. for C_20_H_11_BrN_2_OS: C, 58.98; H, 2.72; N, 6.88%. Found: C, 58.77; H, 2.57; N, 6.69%.

*Antimicrobial activity*

*Microorganisms*

The antifungal activities of the synthetic compounds (5a-i) against fifteen standard strains of fungi including *Candida albicans *(*C. albicans*) (ATCC 10261, 5982, 1912,1905), *Candida dubliniensis* (*C. dubliniensis)* (CBS 8501, ATCC 8500, 7987), *Candida tropicalis* (C*. tropicalis*) (ATCC 750), *Candida krusei* (*C. krusei*) (ATCC 6258), *Candida glabrata* (*C. glabrata*) (ATCC 90030, 863, 2175, 6144), *Candida parapsilosis* (*C. parapsilosis*) (ATCC 4344), *Cryptococcus neoformans *(*C. neoformans*) (ATCC 9011), *Aspergillus flavus* (*A. flavus*) (ATCC 64025), and *Aspergillus fumigates *(*A. fumigates*) (ATCC 14110, CBS 144.89) were determined. The antibacterial activities of the synthetic compounds against standard species of *Staphylococcus aureus *(*S. aureus*) (ATCC 25923, 29213), *Enterococcus faecalis *(*E. faecalis*) (ATCC11700), *Escherichia coli* (*E. coli*) (ATCC 25922), enterohemorrhagic* E. coli* (ATCC 43894), *Pseudomonas aeruginosa *(*P. aeruginosa*) (ATCC 27853) and a clinical isolate of *Shigella flexneri* (*S. flexneri*) collected from the Dr. Faghihi Hospital (Shiraz, Iran) were also determined in this study.

*Determination of minimum inhibitory concentration (MIC)*

The MICs were determined using the broth microdilution method recommended by the CLSI (27-29) with some modifications. Briefly, for determination of antifungal activities, serial dilutions of the synthetic compounds (0.5 to 256 µL/mL) were prepared in 96-well microtitre plates, using RPMI-1640 media (Sigma, St. Louis, USA) buffered with MOPS (Sigma, St. Louis, USA). To determine the antibacterial activities, serial dilutions of the compounds (0.5-256 µL/mL) were prepared in the Muller-Hinton media (Merck, Darmstadt, Germany). For yeasts and bacteria, stock inoculums were prepared by suspending three colonies of the examined microorganisms in 5 mL sterile 0.85% NaCl, and adjusting the turbidity of the inoculums to 0.5 McFarland standard at 630 nm wavelength (this yields stock suspension of 1-5 × 10^6^ cells/mL for yeasts and 1-1.5 × 10^8^ cells/mL for bacteria). The working suspension was prepared by making a 1/1000 dilution of the stock suspension with RPMI or the Muller-Hinton broth for yeasts and bacteria, respectively. For molds (*Aspergillus*spp), conidia were recovered from the 7-day old cultures grown on potato dextrose agar by a wetting loop with Tween 20. The collected conidia were transferred in sterile saline and their turbidity was adjusted to optical density of 0.09 to 0.11 that yields 0.4-5 × 10^6^ conidia/mL. The working suspension was prepared by making a 1/50 dilution with RPMI of the stock suspension. To each well of the microtiter plates, 0.1 mL of the working inoculums was added and the plates were incubated in a humid atmosphere at 30 ºC for 24-48 h (fungi) or at 37 ºC for 24 h (bacteria). Two-hundred microliters (200 μL) of un-inoculated medium was included as a sterility control (blank). In addition, growth controls (medium with inoculums but without the compounds) were also included. The growth in each well was compared with that of the growth control well. MICs were visually determined and defined as the lowest concentration of the compounds produced ≥95% growth reduction compared with the growth control wells. Each experiment was performed in triplicate. 

In addition, media from wells with fungi showing no visible growth were further cultured on Sabouraud dextrose agar (Merck, Darmstadt, Germany) and from wells with bacteria showing no visible growth on the Muller-Hinton agar (Merck, Darmstadt, Germany), to determine the minimum fungicidal concentration (MFC) and minimum bactericidal concentration (MBC), respectively. MFCs and MBCs were determined as the lowest concentration yielding no more than 4 colonies, which corresponds to a mortality of 98% of microorganisms in the initial inoculums.

*Docking simulations*

Molecular docking of compounds 5a-i into the three dimensional X-ray structure of protein tyrosine phosphatase (PDB code: 2OZ5) and 14α-demethylase (PDB code: 1EA1), was carried out using the program AutoDock 4.2 (ADT) (30).

**Figure 1:**
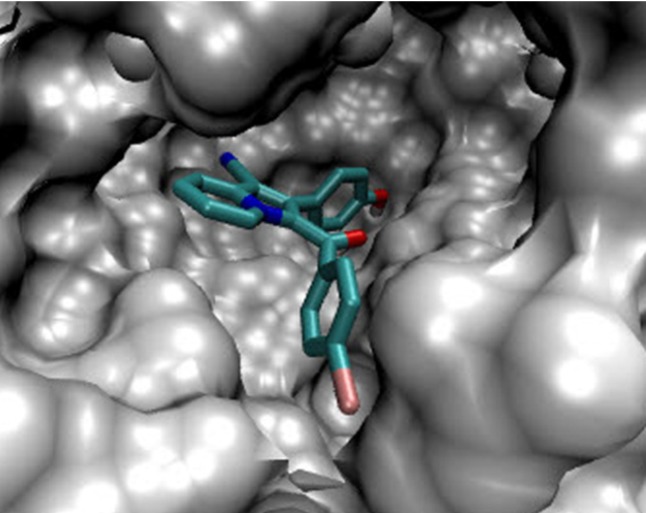
Accomodation of compound 5g in the active site of PtpB (Protein tyrosine phosphataseB).

**Figure 2:**
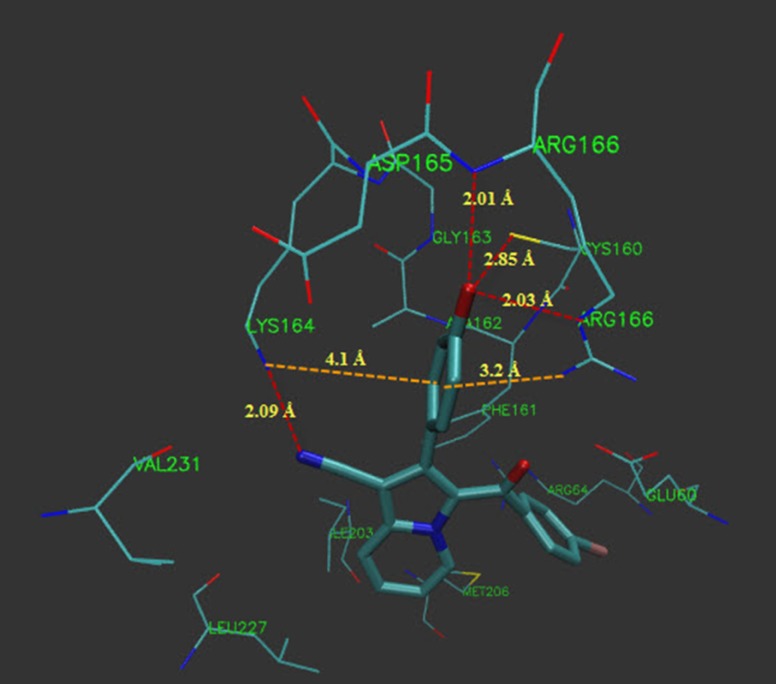
Hydrogen bonds and π-cation interactions of compound 5g with active site residues of PtpB (Protein tyrosine phosphataseB).

**Figure 3:**
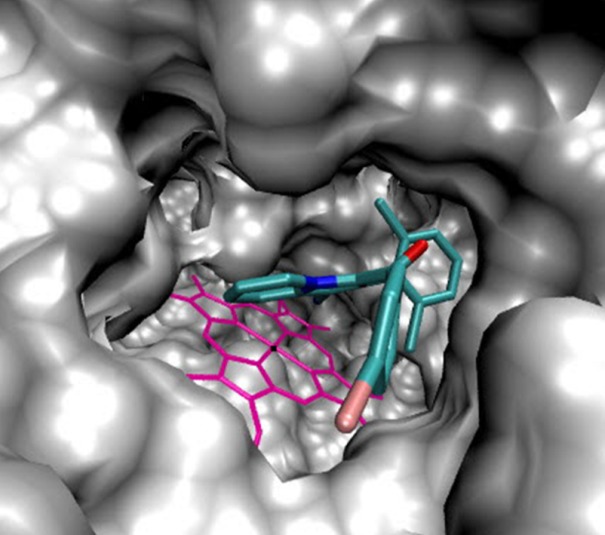
Accomodation of compound 5b in the active site of 14α-demethylase

**Figure 4:**
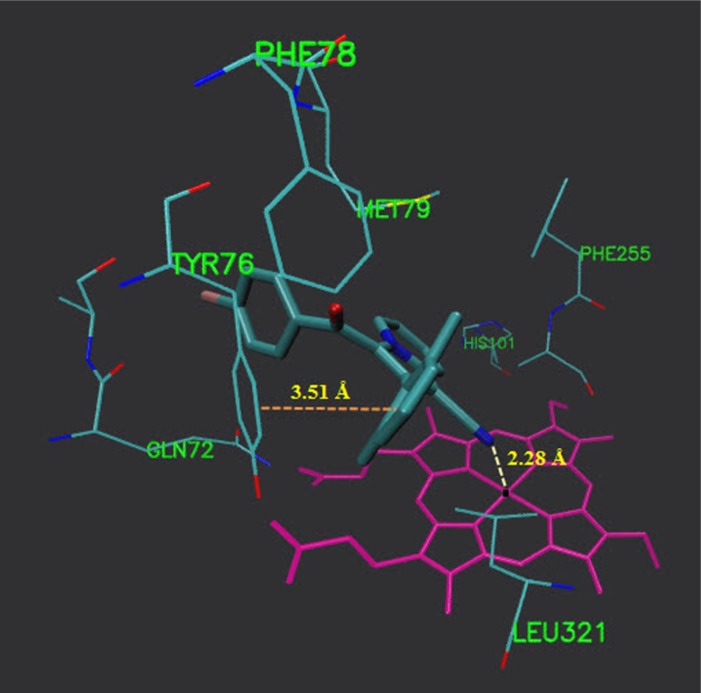
Interaction of compound 5b with active site residues of 14α-demethylase and molecule

The three-dimensional structures of the aforementioned compounds were constructed and energy minimized using the Open Babel 2.3.2 software. The crystal structures of the protein complex were retrieved from the RCSB ProteinData Bank (http://www.rcsb.org). All bound water molecules and ligands were eliminated from the protein and polar hydrogens were added to the proteins. Kollman charges were used for the protein while Gasteiger-Hückel charges were calculated for the ligand. Molecular docking of all nine compounds was then carried out using the AutoDock 4.2 software. The grid box was centered, based on the cognate ligand with a spacing of 0.375 Å and 60 × 60 × 60 dimensions. Parameters of genetic algorithm were set to one hundred runs, 2,500,000 energy evaluations and 150 population size. Cluster analysis was performed on the docked results, using an RMSD tolerance of 2 Å. A VMD molecular viewer was used for creating pose of docking compounds (Figures 1-4).

## Results and Discussion

*Chemistry*

The synthetic route of indolizine-1-carbonitrile derivatives (5a-i) as target compounds is depicted in Scheme 1. Reaction of1-(2-(4-bromophenyl)-2-oxoethyl)-2-chloropyridin-1-ium bromide (4) with arylidenemalonitrile derivatives (3a-i) were carried out in the presence of triethylamine as base and acetonitrile as solvent, under ultrasound irradiation at room temperature. The first step is preparation of 2-chloropyridinium salt (4) which was obtained from the reaction of 2,4′-dibromoacetophenone with 2-chloropyridine according to a literature procedure (31). In the second step, Knoevenagel condensation between aromatic aldehydes (1a-i) and malononitrile (2) took place to form the arylidenemalononitrile derivatives (3a-i). The reaction was performed in acetonitrile under ultrasound irradiation at room temperature for about 5 min. The third step is the 1,3-dipolar cycloaddition of 2-chloropyridinium ylide to arylidenemalononitrile (3a-i), followed by the elimination of HCN and HCl under ultrasound irradiation condition, to yield indolizine-1-carbonitrile derivatives (5a-i) in good yields (32). Both physicochemical and spectral data of all the synthesized compounds were found to be in full agreement with the proposed structures.

*Biological activity*

*Antibacterial activity*

The synthesized compounds were tested for their antibacterial activity against eleven gram-negative bacterial strains and ten gram-positive bacterial strains by broth microdilution method with Muller-Hinton medium. Table 1 summarizes the inhibitory activities of the synthetic compounds against the tested bacteria. In comparing the MIC values of the synthetic compounds, compounds 5h exhibited the best antibacterial activities against gram-positive cocci at concentrations ranging from 16 to 32 µg/mL (Geometric Mean (GM) MIC = 23.7 µg/mL) followed in activity by compounds 5a (GM MIC = 32 µg/mL) and 5g (GM MIC = 35.3 µg/mL), respectively. Beside the bacteriostatic properties, compounds 5a and 5g exhibited bactericidal activities against gram-positive cocci (GM MBC = 86.1 µg/mL). Of the tested compounds, 5a, 5f, 5g and 5h inhibited the growth of gram-negative bacteria at concentrations between 64–256 µg/mL, of which 5g exhibited bactericidal activities with MBCs similar to their corresponding MICs.

**Table 1:** Antibacterial activity (MIC and MBC) of the synthetic compounds (µg/mL).

**Compound**		**MIC**					**MBC**		**Cntl** ‡
		**5a**	**5c**	**5f**	**5g**	**5h**	**5a**	**5g**	
Gram-positive	*S. areus *ATCC 25923	64	G	G	64	32	256	128	0.25
	*S. areus *ATCC29213	64	64	G	64	8	256	128	0.25
	*MRSA1*	64	G	G	64	16	64	128	4
	*MRSA2*	16	G	G	16	16	32	64	16
	*MRSA3*	16	G	G	64	16	256	64	8
	*MSSA1*	32	G	G	64	64	64	64	0.25
	*MSSA2*	32	G	G	8	32	64	128	0.125
	*MSSA3*	32	G	G	32	16	64	64	0.125
	*E. faecalis *ATCC 11700	G	64	G	G	G	G	G	0.5
	*B. cereus *ATCC 11778	G	64	G	G	G	G	G	0.25
Gram-negative	*E. coli *ATCC25922	64	G	128	256	128	G	256	0.06
	TGCR* E. coli1*	G	G	G	G	G	G	G	8
	TGCR* E. coli2*	G	G	G	G	G	G	G	8
	TGCR* E. coli3*	G	G	G	G	G	G	G	16
	TGCS* E. coli1*	G	G	G	G	G	G	G	0.5
	TGCS* E. coli2*	G	G	G	G	G	G	G	0.125
	TGCS* E. coli3*	G	G	G	G	G	G	G	0.25
	*P. aeroginosa *ATCC 27853	G	G	G	G	G	G	G	0.25
	*P. aeroginosa* †	G	G	G	G	G	G	G	16
	*P. aeroginosa* †	G	G	G	G	G	G	G	32
	*P. aeroginosa* †	G	G	G	G	G	G	G	16

MIC: Minimum inhibitory concentration; MBC: Minimum microbicidal concentration; MRSA: Methicillin resistant *S. aureus*; MSSA: Methicillin sensitive *S. aureus*; TGCR *E. coli*: Third generation cephalosporines resistance *E.coli*; TGCs *E.coli*: Third generation cephalosporines sensitive*E.coli.*

* For the compounds 5b, 5d, 5e and 5i no significant antibacterial effect was found.

** "G" stands for Growth (>512 µg/mL).

† Clinical isolates.

‡ Oxacillin was used as control against gram-positive bacteria (*S. aureus, B. cereusc*and *Enterococci *spp), ciprofloxacin and ceftriaxon were used as control against *P. aeruginosa* and *E. coli* species respectively.

**Table 2:** Antifungal activity (MIC and MFC) of the synthetic compounds (µg/mL).

**Compounds**		**MIC**								**MFC**	**Fluconazole** ^†^
		**5a**	**5b**	**5c**	**5e**	**5f**	**5g**	**5h**	**5i**	**5b**	
Yeasts	*C. albicans* ATCC 5982	G	16	32	G	256	64	256	256	256	0.25
	*C. albicans* ATCC 1912	G	16	8	G	128	32	128	256	256	1
	*C. albicans* ATCC 1905	G	36	32	G	G	32	128	128	128	0.25
	*C. dubliniensis* ATCC 8500	128	8	64	G	128	64	256	128	128	0.25
	*C. dubliniensis* ATCC 7987	G	32	128	G	G	64	256	128	128	1
	*C. glabrata* ATCC 2175	G	32	128	G	256	128	256	G	256	0.25
	*C. glabrata* ATCC 90030	G	8	32	G	256	32	G	128	64	0.5
	*C. glabrata* ATCC 6144	G	32	64	G	256	64	256	G	256	0.5
	*C. glabrata* ATCC 863	G	8	128	G	256	64	256	G	256	0.5
	*C. glabrata* ATCC 2175	G	32	128	G	256	128	256	G	256	0.25
	*C. krusei *ATCC 6258	G	32	16	G	256	16	128	256	256	64
	*C. parapsilosis* ATCC 4344	128	8	16	128	128	16	64	128	128	0.25
	*C. tropicalis *ATCC 750	G	16	16	G	256	64	256	256	256	2
	*Cryptococcus neoformans* ATCC 9011	G	G	G	G	G	G	256	G	G	0.25
Filamentous Fungi	*Aspergillusflavus* ATCC 64025	G	32	128	G	G	G	G	G	64	16
	*Aspergillusfumigatus *ATCC 14110	G	32	G	G	G	G	G	G	64	8
	*Aspergillusfumigatus *CBS 144.89	G	128	G	G	G	G	G	G	G	8

* For the compounds 5d no significant antibacterial effect was found.

* "G" stands for Growth (>512 µg/mL).

** MIC: Minimum inhibitory concentration; MFC: Minimum fungicidal concentration.

† Fluconazole was used as control against yeasts and filamentous fungi.

*Antifungal activity*

Table 2 shows the antifungal activities of the synthetic compounds. Among the tested compounds, 5b exhibited the best antifungal activities against all the tested *Candida* species (Geometric mean (GM) MIC_50_ = 16.9 µg/mL, range MICs = 8–32 µg/mL) followed in activity by 5c (GM MIC_50_ = 38 µg/mL, range MICs = 8–128 µg/mL), and 5g (GM MIC_50_ = 45.2 µg/mL, range MICs = 16-128 µg/mL), respectively. None of the tested compounds exhibited antifungal activities against the standard strains of *Aspergillus* except 5b**,** which exhibited fungicidal and fungistatic activities at concentrations of 32 and 64 µg/mL, respectively. None of the tested compounds showed antifungal activities against the standard strain of *Cryptococcus neoformance,* at the examined concentrations. No antifungal activity was found against the fungi at the examined concentrations by 5a, 5d, and 5e. Among the synthetic compounds, only 5b exhibited fungicidal activities against the examined *Candida* and *Aspergillus* species, at concentrations ranging from 64 to 256 µg/mL. In addition, the growth of azole-resistant isolates of *Candida *(*C. krusei, *ATCC 6258) was inhibited by some of the synthetic compounds as shown in Table 2.

*Molecular docking study*

*Docking of synthesized compounds to the active site of Protein Tyrosine Phosphatase (PDB code: 2OZ5)*

Protein phosphorylation and dephosphorylation reactions are employed by living organisms for the regulation of numerous cellular processes, including the growth and differentiation of eukaryotic cells. Protein tyrosine phosphatases (PTPs) are essential for the virulence of several pathogenic bacteria (10). The central roles of PTPs in eukaryotic signaling are exploited by some pathogenic bacteria, which produce and secrete PTPs to attenuate host immune defenses (12). PTP inhibition would thus constitute a valuable strategy against infectious diseases (13). It has been revealed that the phosphotyrosine-binding pockets of different PTP enzymes are similar (33). Some of the 3-substituted indolizine-1-carbonitriles have been shown to have good activity (IC_50 _= 7 µm) against *Mycobacterium tuberculosis* Protein Tyrosine Phosphatase (PtpB) (10). 

With the aim to suggesting the interaction of these compounds with PtpB, molecular docking of thesynthesizedcompounds (5a-i) into the active site cavity of PtpB was performed based on the PtpB complex structure (PDB code: 2OZ5). All compounds were able to accommodate in the active site of the enzyme and the most potent antibacterial compound (5g) was selected for further investigation of interactions.

In the binding mode, compound 5g was nicely bound (ΔG = -9.93 kcal/mol) to the PtpB active site (Figure 1) via four hydrogen bonds together with two π-cation interactions (Figure 2).

The hydroxyl oxygen of 5g formed hydrogen bond with the sulfhydryl hydrogen of Cys 160 (bond length: Cys160 S-H^…^O 2.85 Å; bond angle: Cys160 S-H^…^O = 148°). The nitrogen of the nitrile group of 5g formed hydrogen bond with the amino hydrogen of Lys 164 (bond length: Lys164 N-H^…^N 2.09 Å; bond angle: Lys164 N-H^…^N = 114°). A charge assisted H-bond interaction through the nitrogen of Arg 166 guanidinium group and hydroxyl oxygen of 5g was detected (bond length: N-H^…^O 2.03 Å; bond angle: N-H^…^O = 152°). Another hydrogen bond was also formed by the hydroxyl oxygen of 5g and the amide NH of Arg 166 (bond length: N-H^…^O 2.01 Å; bond angle: N-H^…^O = 166°). A π-cation interaction between the end amino of Lys 164 and hydroxyphenyl ring of 5g was detected. Meanwhile, the guanidinium group of Arg166 formed another π-cation interaction with the hydroxyphenyl ring of 5g (Figure 2).The interactions of compound 5g as well as involved amino acids which had high similarity with the 2OZ5 cognate (OMTS) interactions (Boot *et al.* 2014), suggested that compound 5g probably can be regarded as a potential inhibitor of PtpB. Thus, these results might be helpful for the design and synthesis of compounds with stronger antibacterial activities.

*Docking of synthesized compounds to the active site of 14α-demethylase (CYP51) (PDB code: 1EA1)*

Because of the existence of azole ring as a substructure of indolizinehetrocycle and with the aim to proposing a mechanism for antifungal activity of these compounds, molecular docking of the synthesized compounds (5a-i) into the active site cavity of 14α-demethylase (CYP51) was performed on the binding model, based on the 14α-demethylase and fluconazole complex structure (1EA1.pdb). All compounds were able to accommodate in the active site of the enzyme and the most potent antifungal agent (5b) was selected for further investigation of interactions.

As illustrated (Figure 3), 5b located in the substrate binding site of 14α-demethylase (ΔG = -10.55 kcal/mol). The docking results revealed that in the binding mode, 5b was bound to the 14α-demethylase active site via multiple hydrophobic interactions with Gln 72, Ala 73, Tyr 76, Phe 78, Met 79, His 101, Phe 255, Ala 256 and Leu 321 (Figure 4). The orientation of nitrile towards heme revealed the existence of a coordination bond between the unoccupied orbital on the nitrogen atom of nitrile group and Fe^2+^ of the heme molecule, with length of 2.28 Å (Figure 4). There was also a π-π stacking interaction between Tyr 76 and the chlorine bearing aromatic ring of compound 5b which had a value of 3.51 Å. The non-bonded contacts and position of compound 5b in the active site chamber of 14α-demethylase is in agreement with the active-site residues identified in the original study released for the crystal structure of 14α-demethylase in complex with azole inhibitors (34). Thus, this molecular docking result, along with the biological assay data, suggests that compound 5b probably can be a potential inhibitor of 14α-demethylase. The result of this work might be helpful in the design and synthesis of novel indolizine based 14α-demethylase inhibitors with stronger activities.

## Conclusion

The present study describes the synthesis, structure elucidations, *in-vitro* antibacterial and antifungal activity assay and docking of indolizine derivatives. A series of indolizine-1-carbonitrile compounds in good yields were synthesized and evaluated. All synthesized compounds were evaluated for *in-vitro* antibacterial activity against a number of gram-positive and negative bacteria. The compounds were also evaluated for *in-vitro* antifungal activity against a number of filamentous fungi and yeasts.

Most of them exhibited potent antibacterial and antifungal activities. Compound 5b showed the most potent *in-vitro* antifungal activity and other compounds showed moderate to significant antifungal activity. Compounds 5h exhibited the best antibacterial activities against gram-positive cocci followed in activity by compounds 5a and 5g, respectively. Beside the bacteriostatic properties, compounds 5a and 5g exhibited bactericidal activities against gram-positive cocci. Of the tested compounds, 5a, 5h and 5g inhibited the growth of gram-negative bacteria, of which 5g exhibited bactericidal activities with MBCs similar to their corresponding MICs. The result of this work might be helpful for the design and synthesis of stronger indolizine-based antibacterial and antifungal agents.
